# Immunoporosis: Role of immune system in the pathophysiology of different types of osteoporosis

**DOI:** 10.3389/fendo.2022.965258

**Published:** 2022-09-06

**Authors:** Weidong Zhang, Ruihan Gao, Xing Rong, Siqi Zhu, Yajun Cui, Hongrui Liu, Minqi Li

**Affiliations:** ^1^ Shandong Key Laboratory of Oral Tissue Regeneration and Shandong Engineering Laboratory for Dental Materials and Oral Tissue Regeneration, Department of Bone Metabolism, School and Hospital of Stomatology, Cheeloo College of Medicine, Shandong University, Jinan, China; ^2^ Center of Osteoporosis and Bone Mineral Research, Shandong University, Jinan, China; ^3^ Affiliated Hospital 2, Jinzhou Medical University, Jinzhou, China

**Keywords:** immune cells, inflammatory factors, postmenopausal osteoporosis, senile osteoporosis, diabetic osteoporosis

## Abstract

Osteoporosis is a skeletal system disease characterized by low bone mass and altered bone microarchitecture, with an increased risk of fractures. Classical theories hold that osteoporosis is essentially a bone remodeling disorder caused by estrogen deficiency/aging (primary osteoporosis) or secondary to diseases/drugs (secondary osteoporosis). However, with the in-depth understanding of the intricate nexus between both bone and the immune system in recent decades, the novel field of “Immunoporosis” was proposed by Srivastava et al. (2018, 2022), which delineated and characterized the growing importance of immune cells in osteoporosis. This review aimed to summarize the response of the immune system (immune cells and inflammatory factors) in different types of osteoporosis. In postmenopausal osteoporosis, estrogen deficiency-mediated alteration of immune cells stimulates the activation of osteoclasts in varying degrees. In senile osteoporosis, aging contributes to continuous activation of the immune system at a low level which breaks immune balance, ultimately resulting in bone loss. Further in diabetic osteoporosis, insulin deficiency or resistance-induced hyperglycemia could lead to abnormal regulation of the immune cells, with excessive production of proinflammatory factors, resulting in osteoporosis. Thus, we reviewed the pathophysiology of osteoporosis from a novel insight-immunoporosis, which is expected to provide a specific therapeutic target for different types of osteoporosis.

## 1 Introduction

Osteoporosis is the most common skeletal disease characterized by low bone mass and microarchitectural alteration of bone tissue, accompanied by the increased risk of fractures (1). The causes of osteoporosis are divided into primary (postmenopausal and senile osteoporosis) and secondary (diabetic osteoporosis, glucocorticoid-induced osteoporosis) causes. The burden of osteoporosis affects patients’ quality of life, mainly due to femur and vertebrae fractures (1). Due to the aging population, osteoporosis incidence is rapidly increasing and gradually becoming a public health problem.

Physiologically, the skeletal system undergoes an orderly and coupled process called bone remodeling (2). Bone remodeling begins with the absorption of mineralized bone by osteoclasts and follows by the osteoblast-mediated formation of bone matrix that becomes mineralized in succession. However, the process of bone remodeling is affected by physiological alterations, including estrogen deficiency, aging, diseases and drugs. Once the balance of bone remodeling breaks, specifically, when the process of bone resorption takes over bone formation, it results in bone loss and ultimately leads to osteoporosis (1). Although classical theories define osteoporosis as an endocrine disease (3), many studies reported that interactive communication exists between skeletal and immune systems in osteoporosis. During the past two decades, a novel interdisciplinary field “osteoimmunology” was established to explore the intricate relationship between the skeletal and immune systems (4). Osteoblast and osteoclast activities are regulated by various soluble mediators secreted from immune cells, including cytokines, chemokines, and growth factors. In reverse, osteoblasts and osteoclasts regulate the hematopoietic stem cell niche from which immune cells are derived (5). Recently, accumulating evidence demonstrated that both innate and adaptive immune cells contribute to the pathogenesis of osteoporosis by producing pro-inflammatory mediators (6, 7). The term “immunoporosis” was proposed and coined by Srivastava et al. (2018, 2022) to establish a novel field emphasizing the role of immune cells in the development of osteoporosis (8, 9).

This review will present the relationship between immune cells and bone remodeling. More importantly, we focused on the pathophysiology of different types of osteoporosis using the novel insight-immunoporosis. This review helps to understand the pathogenesis of different types of osteoporosis from the level of immune cells and is expected to provide a specific therapeutic target for different types of osteoporosis.

## 2 Bone remodeling and immune cells

**Figure 1:**
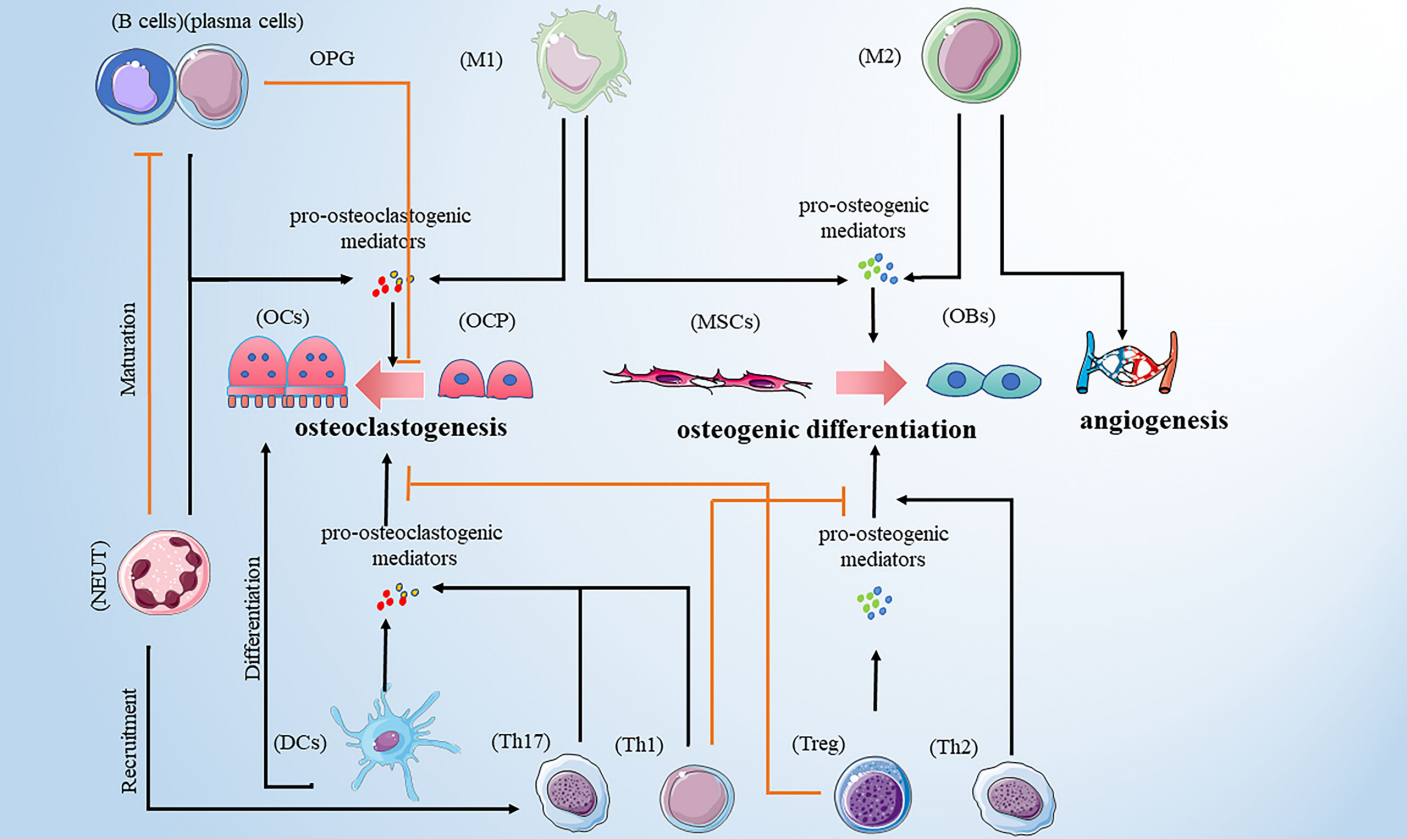
Immune cells promote osteoclastogenesis by secreting pro-osteoclastogenic mediators. Those immune cells include M1 macrophages, DCs, neutrophils, Th1, Th17 and B cells. M1 macrophages are also involved in osteogenic difference. M1 macrophages and Treg cells promote osteogenic differences by releasing pro-osteogenic mediators. Th2 cells play a bone-protective role. In addition to promoting osteoclastogenesis, neutrophils can recruit Th17cells and inhibit the maturation of B cells. B cells play a dual role in osteoclastogenesis. Note: Various types of immune cell image materials are from https://smart.servier.com.

Bone remodeling is a dynamic and continuous process that maintains skeletal health (10). The process involves three consecutive phases: osteoclasts-mediated resorption; reversal, during which mesenchymal derived osteoblasts are recruited to the bone site of bone resorption; and osteoblasts-mediated formation. Hence, osteoblasts and osteoclasts are two major players in bone remodeling. However, multiple pro-osteoclastogenic and pro-osteogenic mediators are released by innate and adaptive immune cells influencing bone cell function (**Table 1**). In addition, different types of bone cells affect the activity of immune cells, and their complex interactions form a complex bone microenvironment. Therefore, we reviewed the relationship between common immune cells and bone remodeling (**Figure 1**).

**Table 1:** The pro-osteoclastogenic and pro-osteogenic mediators secreted by immune cells.

Cells	pro- osteoclastogenic mediators	pro-osteogenic mediators	Reference
macrophages	TNF-α, IL-1β, IL-6, ROS	TGF-β, IGF-1, CCL2, CCL5	(11, 12)
Dendritic cells	IL-1, IL-6, IL-7, IL-15, TNF-α	–	(13, 14)
neutrophils	IL-17, RANKL	FGF-2, PDGF, TGF-β	(15–17)
T cells	TNF-α, RANKL, IFN-γ, IL-1, IL-6, IL-17, IL-22	IFN-γ, IL-10, TGF-β, IL-17	(18–20)
B cells	RANKL	–	(21)

### 2.1 Innate immune cells

#### 2.1.1 Macrophages

Macrophages derive from the monocytic lineage, which performs immune sentinel and homeostatic functions by recognizing and eliminating pathogenic organisms. Macrophages play an essential role in the recruitment and activation of other immune cells, including T lymphocytes (T cells). Furthermore, macrophages maintain immune homeostasis by transforming polarized phenotypes (22). They infiltrate tissues during inflammation, forming pro-inflammatory phenotypes (M1) and anti-inflammatory phenotypes (M2) in different immune microenvironments. M1 macrophages are polarized by lipopolysaccharide (LPS) either alone or accompanied by T-helper 1 (Th1) cytokines, such as Interferon-γ (IFN-γ), and have the pro-inflammatory effect by producing cytokines, including interleukin-1β (IL-1β), IL-6, IL-12 and tumor necrosis factor-alpha (TNF-α). Under the stimulation of T-helper 2 (Th2) cytokines, such as IL-4 and IL-13, M2 macrophages paly an anti-inflammatory and immunoregulatory role by secreting anti-inflammatory cytokines, such as IL-10 and TGF-β. The prevailing effect of M1 macrophages is the promotion of osteoclastogenesis with a high level of reactive oxygen species (ROS) (23) and pro-osteoclastogenic cytokines, including TNF-α and IL-1β (11). In addition, a study by Liang B et al. found that M1 macrophages can promote osteogenesis by secreting high levels of chemokines to recruit mesenchymal stem cells (MSCs) (11), while another study found that M1 macrophages could promote osteoblast differentiation *via* cyclooxygenase-2 (COX-2)-prostaglandin E2 (PGE2) pathway (24). In contrast, M2 macrophages have a bone-protective role (22) and promote bone mineralization by stimulating MSCs and precursor osteoblasts by differentiating into mature osteoblasts. On the other hand, M2 macrophages have a high angiogenic potential, indirectly promoting osteogenesis. Additionally, M2 macrophage subsets participate in apoptotic cell clearance, contributing to steady-state bone turnover. A recent study by Kalluri R et al. found that extracellular vesicles (EVs), which contain protein and micro-RNA cargo, are secreted by some cells and endocytosed by target cells whose function is affected by those EVs’ cargo (25). Although macrophage-derived EVs contain different types of miRNAs, the current mainstream view is that naïve (M0) and M2-derived EVs promote repair/regeneration and M1 EVs inhibit bone repair and promote bone loss (26, 27). Interestingly, mesenchymal stem cell-derived EVs affect the activity and polarization of macrophages (28, 29), suggesting the interaction between mesenchymal stem cells and immune cells. The balance of M1/M2 macrophage polarization governs the fate of an injured or inflamed organ. Similarly, the phenotypic switch between M1 and M2 macrophage populations is”fluid” rather than “fixed” in response to the local bone microenvironment, which is closely related to bone remodeling (12, 22). Hence, the relationship between macrophages and bone remodeling attracts more attention.

#### 2.1.2 Dendritic cells

DCs are the primary antigen-presenting cells derived from monocyte/macrophage progenitor cells and can activate adaptive immune responses. In addition to the exceptional ability to present antigens, DCs play distinct roles in regulating T lymphocytes cells development, differentiation, and function. Current evidence shows that DCs critically contribute to the differentiation and homeostasis of Treg cells, which serve an essential role in promoting osteogenesis and inhibiting osteoclastogenesis *via* anti-inflammatory effect (30). However, DCs express receptor activator of NF-kappaB (RANK) expressed in osteoclasts. Additionally, DCs also produce pro-inflammatory cytokines to promote the formation of osteoclasts, including IL-1, IL-6, and TNF-α. A recent study confirmed that in the presence of the receptor activator of NF-kappaB ligand (RANKL), DCs could transdifferentiate into osteoclasts and involve bone resorption (13). Newly formed osteoclasts can induce chemotaxis of DCs to call on more DCs, creating an osteoclast-DC cycle that continues to increase bone destruction (13). This evidence suggests that DCs serve a dual role in bone remodeling and has great potential for further research.

#### 2.1.3 Neutrophils

Neutrophils, an effector of the innate immune response, originate from hematopoietic precursors in the bone marrow and are recruited into infected tissue to neutralize pathogens by releasing proteases and toxic enzymes. Neutrophils are reported to have protective functions for bone formation at the early stages of bone healing. A review of the role of neutrophils infiltrated that neutrophils can secrete many pro-angiogenic growth factors and osteogenic factors, such as fibroblast growth factor-2 (FGF-2), platelet-derived growth factor (PDGF), and transforming growth factor beta (TGF-β) (15). However, the hyperactive neutrophils triggered by infection or injury can harm bone homeostasis. Hajishengallis G et al. argued that neutrophils could produce chemokines to recruit proinflammatory cells, such as Th17 cells, and suppress B lymphocytes, thereby promoting inflammatory bone loss (16). In addition, Chakravarti A et al. confirmed this idea using *in vitro* experiments and found that activated neutrophils secrete RANKL, contributing to the formation and maturation of osteoclasts (17). According to the available evidence, the role of neutrophils on bone remodeling depends on the microenvironment, but the mechanism remains unclear, which needs to be explored further.

#### 2.1.4 Another innate immune cells

Mast cells (MCs) are derived from hematopoietic stem cells in bone marrow, and MCs participate in the regulation of bone homeostasis and the pathogenesis of bone diseases through mediators such as synthase and cytokines. In the study about the osteogenic function of MCs in patients with rheumatoid arthritis, Kim KW et al. found that MCs could directly stimulate osteoclast formation or indirectly produce tissue-destroying cytokines (31). Natural killer (NK) cells are cytotoxic lymphocytes of the innate immune system that develop from hematopoietic stem cells. NK cells induce differentiation of osteoclasts in an M-CSF and RANKL-dependent manner to regulate bone remodeling (32). Moreover, through the *in vitro* validation, Feng S et al. found that cytotoxic NK cells could control the pathogenic bone resorption of osteoclasts (33). However, other studies have shown that IL-15-activated NK cells could kill osteoclasts and inhibit bone erosion, which requires contact between NK cells and osteoclasts (34). Therefore, NK cells can control or increase osteoporosis depending on the tissue microenvironment.

### 2.2 Adaptive immune cells

#### 2.2.1 T lymphocytes (T cells)

T cells originate from bone marrow and mainly mature in the thymus, including two prominent cell families: CD4^+^ (helper) and CD8^+^ (cytotoxic) groups. T cell-mediated cellular immune response plays a vital role in inflammatory diseases, which can directly kill target cells by specifically binding and disrupting the membrane or releasing lymphokines that amplify and enhance the immune effect.

CD4^+^ T cells interact with other immune cells by surface receptors and secret cytokines to increase or decrease their activation state (35). According to their cytokine expression profile, CD4^+^ T cells are divided into different subsets, including Th1, Th2, Th9, Th17, Th22, Tfh, and Treg cells. Th1, Th2, Th17, and Treg cells are differentiated from Th0 cells as the main effector cells. Th1 cells are involved in the cell-mediated response to local inflammation, and they can secrete cytokines that act mainly on macrophages to exert pro-inflammatory effects. Trigged by IL-12, Th1 cells produce IFN-γ and TNF-α and can stimulate macrophage polarization toward the M1 phenotype. Previously, Th1 cells were thought to be primarily involved in inflammatory bone loss (36). However, later evidence found that Th1 cells may play a dual role in osteoclastogenesis due to the effect of IFN-γ. On the one hand, IFN-γ increases the degradation of ubiquitin ligase TRAF6, inhibiting the formation of osteoclasts. Further, IFN-γ promotes osteoclast maturation in the late stage of osteoclast formation (18). Additionally, Th1 cells are also the producers of TNF-α, which are reported to increase osteoblast apoptosis and promote osteoclastogenesis by increasing the expression of RANKL (37), and the mechanism of action needs to be explored further. Usually, Th2 cells are involved in the humoral immune process and assist in activating B cells, which play an anti-inflammatory role. In addition, Th2 cells are characterized by the production of IL-4, IL-5, and IL-13; they also participate in macrophage polarization to the M2 phenotype. There is some evidence for the relationship between Th2 cells and osteoclasts. The available evidence suggests that Th2 cells may serve a bone-protective role. A review by Pacifici R et al. concluded that active Th2 cells maintain osteoblast activity by enhancing the production of parathyroid hormone (PTH) (38). However, there are fewer relevant studies on its role in bone metabolism, specifically about the relationship between Th2 cells and osteoclasts. Th17 cells are essential in developing autoimmune diseases by secreting various pro-inflammatory factors, participating in the development of inflammation, and enhancing immunopathological damage. It can promote osteoclastogenesis by secreting various pro-inflammatory cytokines, including IL-1, IL-17, IL-22, and TNF-α (19). The prevailing view was that IL-17 upregulates the RANK receptor on osteoclast precursors to promote osteoclast differentiation (39). However, its role in osteoblasts is still controversial. Conversely, IL-17 can inhibit BMP-2-induced osteoblast differentiation (40) and induce pyroptosis in osteoblasts through the NLRP3 inflammasome pathway *in vitro* (41). Recent studies have reported that IL-17 can promote osteoblast differentiation, bone regeneration, and remodeling in mice (42). These different accounts provide clues to the IL-17 role in bone metabolism and deserve further investigation. Treg cells are a subset of cells with broad immunosuppressive and immunomodulatory effects. Producing anti-inflammatory cytokines, including IL-10 and TGF-β, can inhibit the over-activation and proliferation of many immune cells in the body, weaken the inflammatory response and maintain the stability of the immune system. A prevailing view pointed out that Treg cells can directly suppress the maturation of osteoclasts by expressing cytotoxic T lymphocyte-associated antigen-4 (CTLA-4) to remove the costimulatory molecule, CD80/CD86 expressed on osteoclast precursors (43). However, a recent study reported that Treg cells have a bone-protective effect by reducing osteoclast numbers rather than causing an intrinsic defect in osteoclast differentiation (44). However, these ideas are not yet fully developed, and the role of Treg on osteoblasts is still unclear, which needs to be further explored. Accordingly, the balance of Th1/Th2 and Th17/Treg cells is crucial in maintaining bone homeostasis in the physiological state. Once pathogenic factors cause the disorder in Th1/Th2 and Th17/Treg balance, the bone remodeling process is bound to be affected, resulting in various bone diseases.

CD8^+^ T cells serve an important role in the clearance of intracellular pathogens and emergent neoplasms, and the mature CD8^+^ T cell is known as a cytotoxic T cell because of its role in recognizing damaged somatic cells and triggering the death pathway through cytotoxic proteins. They can kill target cells and enhance T cell-target cell interactions specifically. It has been reported that CD8^+^ T cells have an inhibitory effect on osteoclastogenesis by secreting various soluble proteins, such as osteoprotegerin (OPG), a soluble RANKL decoy receptor, to suppress the interaction of RANKL-RANK (45). In addition, osteoclasts and CD8^+^ T cells can form a negative feedback loop, contributing to the homeostasis of the skeletal and immune systems, which play a protective role in bone resorption (46). It has been shown that osteoclasts from peripheral blood mononuclear cells can activate CD8^+^ T cells (47). These CD8^+^ T cells are shown to be noncytolytic and anergic, expressing CD25 and Foxp3, therefore referred to as osteoclast-induced regulatory CD8^+^ T cells or OC-iTcREG. OC-iTcREG can express membrane-bound RANKL and CTLA-4 and produce IFN-γ, IL-6, IL-10, and IL-2 (48, 49). These factors can have either positive or negative effects on osteoclasts, thus allowing CD8^+^ T cells to play a regulatory role in the process of bone remodeling. However, the role of CD8^+^ T cells on osteoblasts is unclear and needs to be explored further. In addition, different types of bone cells have different effects on T cells. There is evidence that osteoblasts support the differentiation of T cells in the bone marrow. A study by Yu VW et al. confirmed that the expression of Notch ligand delta­like 4 by osteoblasts contributes to supporting the development of T cell progenitor (50). Studies on osteoclasts have revealed that antiresorptive drugs can affect the activity of immune cells by inhibiting osteoclast activity. For example, osteoclast precursor cells were shown to have the ability to inhibit T cell proliferation in a mouse model of autoimmune arthritis (51). This evidence suggests a regulatory role of bone cells on T cells, although it is not sufficient and needs to be further explored.

#### 2.2.2 B lymphocytes (B cells)

B cells are derived from pluripotent stem cells in the bone marrow and are known for producing antibodies in adaptive immune responses. B cells can be stimulated by antigens and subsequently proliferate or differentiate into a large number of plasma cells, which secrete antibodies that play an immune clearance role in blood circulation. Further, as antigen-presenting cells, B cells can directly activate T cells and macrophages, thus playing an immunomodulatory role (52). It was reported that both B cells and B-cell-derived plasma cells could regulate osteoclastogenesis by delivering RANKL (21). However, the B cell is also a major source of osteoprotegerin (OPG). In addition, the effect of B cells is influenced by T cell subsets. When activated by Th1 cytokines, B cells can inhibit osteoclastogenesis, but under the stimulation of Th2 cytokines, B cells promote osteoclastogenesis (53). Although current attention to B cells is paid to osteoclastogenesis, a recent study found that B cells inhibited osteoblast differentiation by activating extracellular signal-regulated kinase (ERK) and nuclear factor-kappaB (NF-κB) signaling pathways (54), which resulted in the inhibition of bone formation. However, the study has also shown that mTORC1 signaling in pre-osteoblasts is required for normal B cell development in mice, which means that there may be a bidirectional interaction between B cells and osteoblasts (55). While many aspects of B cell biology in bone remodeling remain unclear, B cell is emerging as one of the key regulators of the process. Apart from that, bone cells affect the activity and function of B cells. Greenbaum A et al. found that depletion of CXCL12 in osteoblasts reduced the number of B lymphoid progenitors in the bone marrow. Therefore, it demonstrated that osteoblasts could support the differentiation of B cells in the bone marrow (56). Furthermore, as a potent inhibitor of osteoblast formation produced by osteocytes, sclerostin deficient mice exhibited high bone mass and reduced mature B cells, suggesting that the regulation of B cells by osteocytes is involved in the bone remodeling process (57). This evidence suggests an inextricable link between the immune and skeletal systems, and thus the concept of the interdependence of the two systems must be considered when exploring disease mechanisms or therapeutic strategies (58).

## 3 Postmenopausal osteoporosis

**Figure 2:**
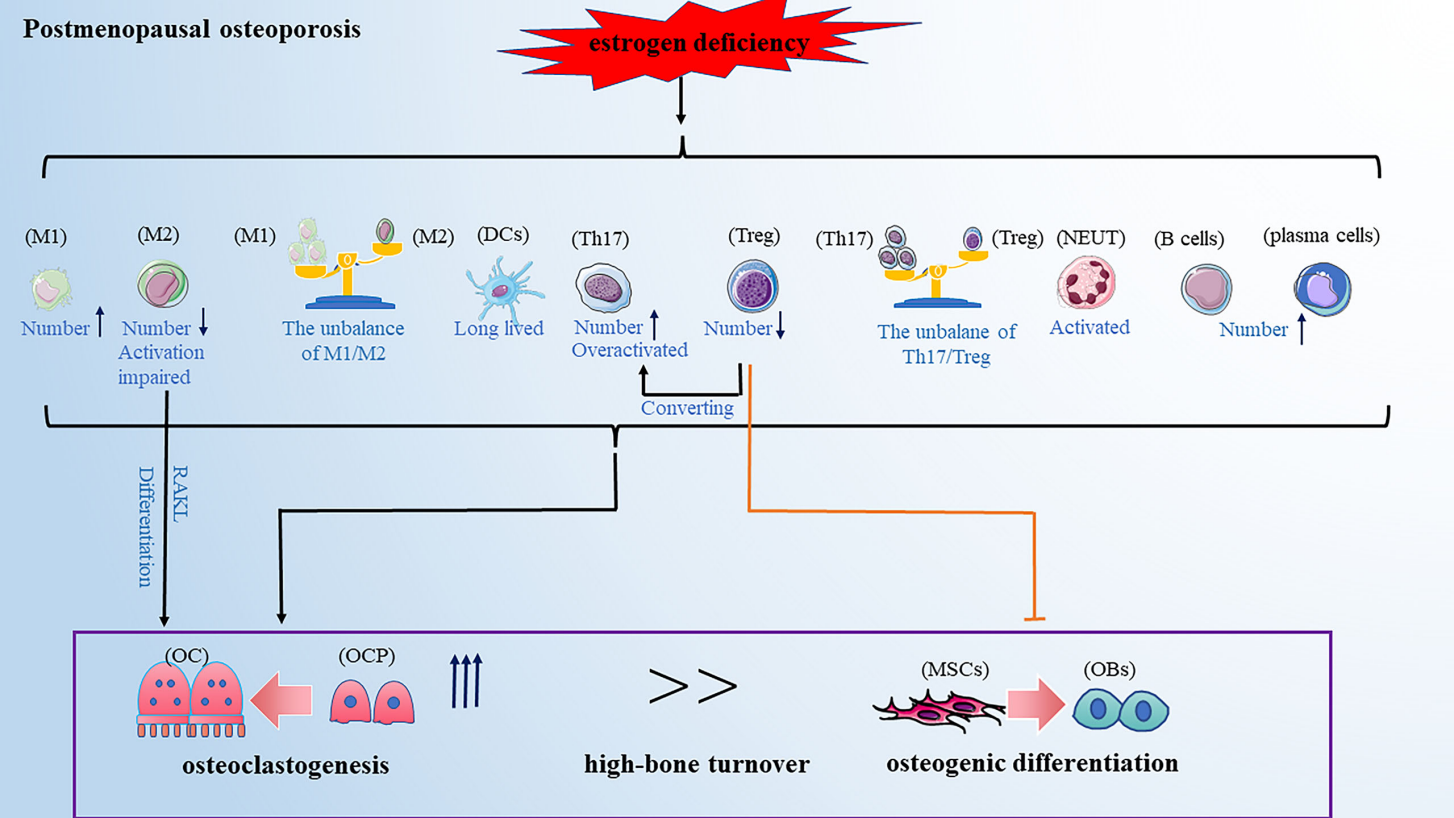
PMOP is a high-bone turnover disease. Estrogen deficiency leads to changes in the number and function of immune cells, which ultimately affecting bone remodeling and leads to osteoporosis. These changes included: the balance of M1/M2 and Th17/Treg favors pro-inflammatory M1 and Th17 cells; the activation of M2 macrophages and Treg cells are impaired; Th17 cells are overactivated and Treg cells can convert into Th17 cells; DCs become long-lived; neutrophils are activated; The number of B cells and plasma cells is increased. Note: Various types of immune cell image materials are from https://smart.servier.com.

Postmenopausal osteoporosis (PMOP) is prevalent in primary osteoporosis and is characterized by excess osteoclastogenesis leading to net bone loss and brittle fractures. A previous WHO report showed that the risk of osteoporotic fractures in postmenopausal women is at least 30% and even closer to 40% (59), suggesting that the health of middle-aged and older women is affected by PMOP-related bone injury which has become one of the urgent clinical problems to be solved. It was reported that PMOP is a high-bone turnover disease: bone resorption is increased, while bone formation is also increased, just not keeping up with the bone resorption (60). The classical theory defined estrogen deficiency as a primary pathogenetic factor in PMOP. Estrogen is shown to have a protective effect on bone resorption by inducing osteoclasts apoptosis and blocking the maturation of osteoclasts (61, 62). However, without the protective effect of estrogen, the balance of bone remodeling favors bone resorption, resulting in PMOP (63). A recent study reported that estrogen deficiency-mediated bone loss has a complex mechanism mainly involving the immune system rather than a mere direct effect of estrogen on bone cells (64). Next, we reviewed the role of estrogen deficiency-mediated alteration of various immune cells on PMOP development (**Figure 2**).

### 3.1 Innate immune cells and PMOP

#### 3.1.1 Macrophages

An ovariectomy (OVX) animal experiment demonstrated that estrogen deficiency made the M1 polarization enhanced and the M2 polarization impaired (65). Further, an increased level of pro-inflammatory cytokines was observed in postmenopausal patients, such as TNF-α and IL-1β (66). Accordingly, M1 macrophages have a promotive effect on osteoclastogenesis, which gives a reason for bone resorption in PMOP [8]. Meanwhile, blunted M2 activation leads to bone loss in PMOP (67). Contrary to our knowledge, M1 macrophages are the potential precursors of osteoclasts, and the researchers found that M2 macrophages can differentiate into osteoclasts without estrogen protection in the presence of RANKL (65). Although M2 macrophages are well known to promote osteogenesis, there is no direct evidence to confirm the relationship between estrogen deficiency-mediated alteration of M2 macrophages and impaired osteogenesis; thus, providing a view to exploring the role of macrophages in PMOP. Recently, the role of residual tissue residual macrophages in bone, osteal macrophages, has received more attention (68). Osteal macrophages are shown to support osteoclast-mediated resorption by scavenging degraded bone byproducts, inflecting that residual tissue macrophages also play an essential role in the pathology of PMOP (69).

#### 3.1.2 DCs and neutrophils

In the absence of estrogen, DCs will long live with increased expression of IL-7 and IL-15. IL-7 and IL-15 induce IL-17 and TNF-α production in a subset of memory T cells, independent of antigen activation (14). These pro-inflammatory cytokines contribute to inflammation-mediated bone loss in PMOP by activating low-grade inflammation. The neutrophil, ratio is considered a helpful clinical tool in assessing PMOP due to its strong association with bone mineral density (70). Accordingly, hyperactive neutrophils favor osteoclastic bone resorption. The *in vitro* assays confirmed that estrogen blocks inflammatory-induced neutrophil overactivation (71), suggesting estrogen deficiency may cause neutrophil activation, which is important in PMOP development.

### 3.2 Adaptive immune cells and PMOP

#### 3.2.1 T cells

Physiologically, thymic output and peripheral consumption contribute to the maintenance and renewal of the T cells in peripheral blood. A recent clinical cross-sectional study demonstrated that compared with premenopausal women, the leukocyte count is elevated in postmenopausal women, reflecting increased total lymphocytes and monocytes (72). Consistently, previous experimental data showed that estrogen deficiency increases the thymus output of T cells in peripheral blood (73). Further analysis found that T cells are overactivated under estrogen deficiency, particularly CD4^+^ T cells. Although the different subtypes of T cells may play a role in promoting or inhibiting bone resorption, it is well recognized that activated T cells contribute to osteoclastogenesis by strongly expressing RANKL in PMOP (74). As an osteoclastogenic subset of T cells, the Th17 cells population was found to be increased in bone marrow, accompanied by the elevated IL-17 level in peripheral blood. Blocking the IL-17 pathway had an effective protective role in bone loss in OVX mice (75). These results suggested that Th17 cells are a potent mediator in PMOP. In contrast, Treg cells have a bone-protective role in PMOP development (76). More importantly, it was reported that Th17/Treg balance is disturbed under estrogen deficiency, enhanced Th17, and decreased Treg cells (77). Tregs cells may lose their immunosuppressive function under estrogen deficiency and convert to Th17 cells, which explains the unbalance of Th17/Treg in PMOP (77).

#### 3.2.2 B cells

The role of B cells is also of interest in PMOP development. Studies found that estrogen deficiency causes an increase in B cells number in the bone marrow, and some of the increased B cells give rise to osteoclasts (78). It was also supported that in OVX mice, estrogen deficiency selectively stimulated the accumulation of B cell precursors, while in the presence of estrogen, the stromal cell-dependent B cells were greatly inhibited (79). In addition, B cells isolated from the bone marrow of postmenopausal women have been reported to secrete RANKL, contributing to osteoclastogenesis (80). These results suggested that estrogen deficiency can directly promote osteoclast formation through stimulating B cells. However, an OVX animal experiment reported that the bone loss in mature B cell-deficient mice was the same as that of wild-type (WT) control mice, suggesting B lymphocytes may not be the central mediators of ovariectomy-induced bone loss (81).

## 4 Senile osteoporosis

**Figure 3:**
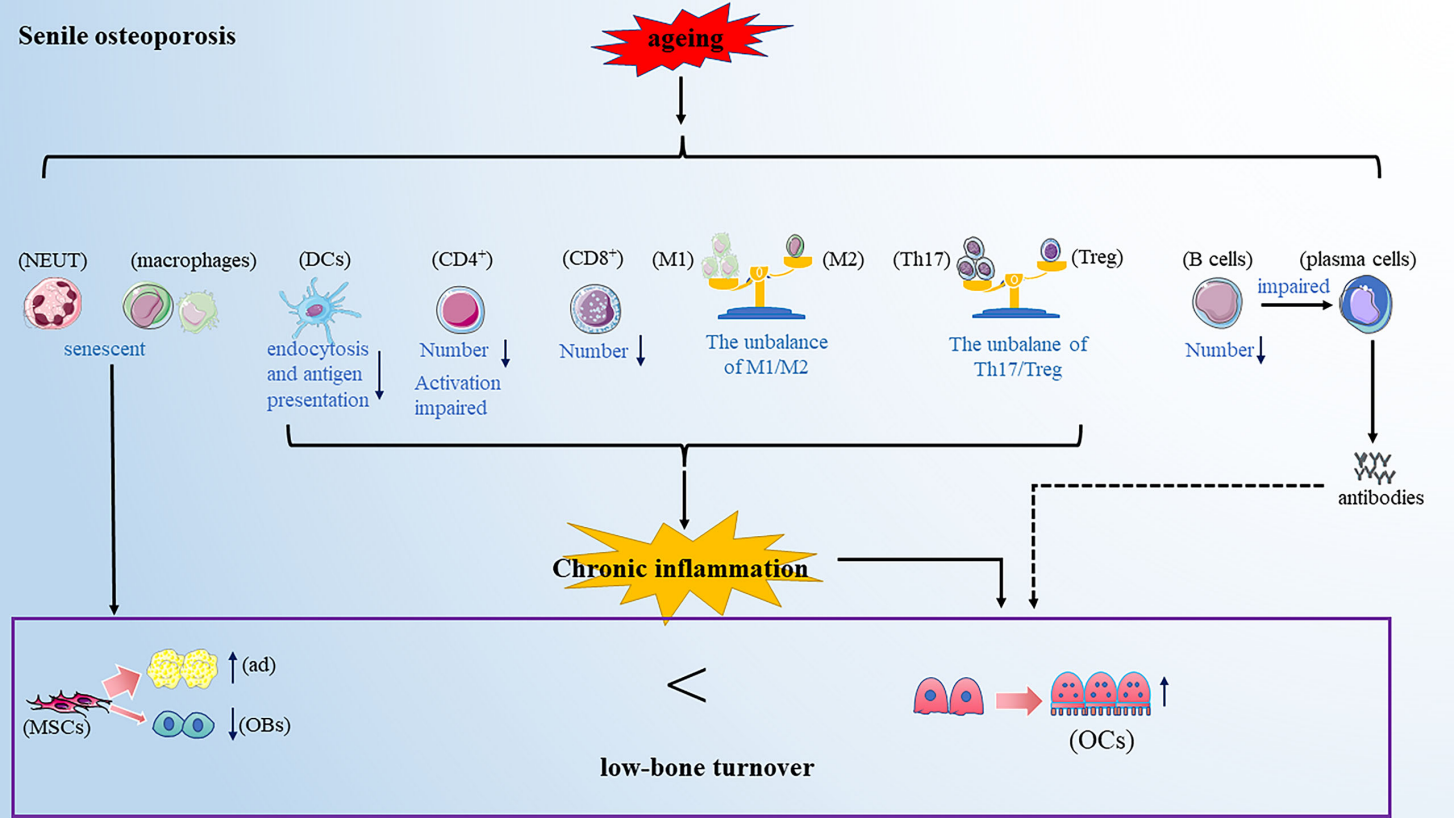
Senile osteoporosis is a low-bone turnover disease. Aging makes the immune cells senescent. On the one hand, the senescent immune cells promote the differentiation of mesenchymal cells into adipocytes and inhibit the differentiation of mesenchymal cells into osteoblasts; further, the altered immune cells promote osteoclastogenesis. Note: Various types of immune cell image materials are from https://smart.servier.com.

Senile osteoporosis commonly occurs in older people above 70s and has become a worldwide health concern with the rising aging world population. Senile osteoporosis is usually described as a low-bone turnover disease with decreased resorption and significantly reduced bone formation (82). However, in recent years, a careful observation found that aging is usually accompanied by systemic low-grade chronic inflammation and enhanced inflammatory mediators, such as IL-6 and TNF-α (83). This will provide an important view of the development of senile osteoporosis (**Figure 3**).

### 4.1 Innate immune cells and senile osteoporosis

#### 4.1.1 Macrophages, neutrophils, and DCs

A recent study found that senescent immune cells, such as macrophages and neutrophils, accumulate in bone marrow during aging in rats and mice (84). The senescent macrophages and neutrophils repress osteogenesis by promoting bone marrow mesenchymal stromal cell adipogenesis. In addition to directly inhibiting osteogenesis, the senescent immune cells contribute to chronic inflammation, thus leading to inflammatory bone resorption. The M1/M2 macrophage polarization balance favors the pro-inflammatory M1 polarization phenotype in old mice (85). Moreover, neutrophil proportions increased and became long-lived and hypersegmented in elder mice, and the persistently low level of increased neutrophils contributes to chronic inflammation by releasing pro-inflammatory TNF-α during aging (86). Additionally, the endocytosis and antigen presentation capacity of DCs diminishes with aging, which may affect the production of T cell-specific cytokines (87), and thus participate in the inflammatory response and osteoporosis process. In summary, the alteration of innate immune cells in the number or function may be one of the mechanisms of senile osteoporosis.

### 4.2 Adaptive immune cells and senile osteoporosis

#### 4.2.1 T cells

The data from experimental models found significant defects in CD8^+^ and CD4^+^ T cell responses with aging (88). The diversity of CD8^+^ T cells was reduced and severely limited the initiation of effective immune responses, leaving in a prolonged state of chronic inflammation (89). In addition, the activation of CD4^+^ T cells was impaired due to aging-induced alteration in cell surface glycosylation and key signaling molecules (90, 91). It has been reported that the unbalance of CD4^+^ T cell subsets may be responsible for chronic inflammation (72). Aging could tilt the balance of Th1/Th2 toward Th2 cells, resulting in an increased inflammatory response (92). Besides, by analyzing the proportion of Th17 and Treg cells in four different age groups from healthy human donors, Vanessa et al. found that the ratio of Th17/Tregs appeared to increase with aging, which may lead the immune system into a hypo-activated state with a high production of pro-inflammatory cytokines (93). In summary, aging contributes to continuous chronic inflammation at a low level by impairing the function of T cells or breaking immune balance, ultimately resulting in bone loss.

#### 4.2.2 B cells

It has been widely demonstrated that aging greatly influences the number and function of B cells. A significant reduction of circulating B cells was observed in the aged bone marrow microenvironment, primarily due to the decreased formation of B cells in bone marrow (94). Besides, the elderly showed the impaired ability of memory B cells to differentiate into plasma cells and produce high-affinity protective antibodies against newly encountered antigens (95). It allows healthy elderly individuals with chronic diseases to share similar features of B cells impairment, such as loss of protective immunity and poor response to vaccinations (96). All this evidence is closely related to the previously described relationship between advanced age and chronic systemic inflammation. However, there was a lack of direct evidence on the relationship between aging-induced B cells dysfunction and osteoporosis, so further research is needed.

## 5 Diabetic osteoporosis

**Figure 4:**
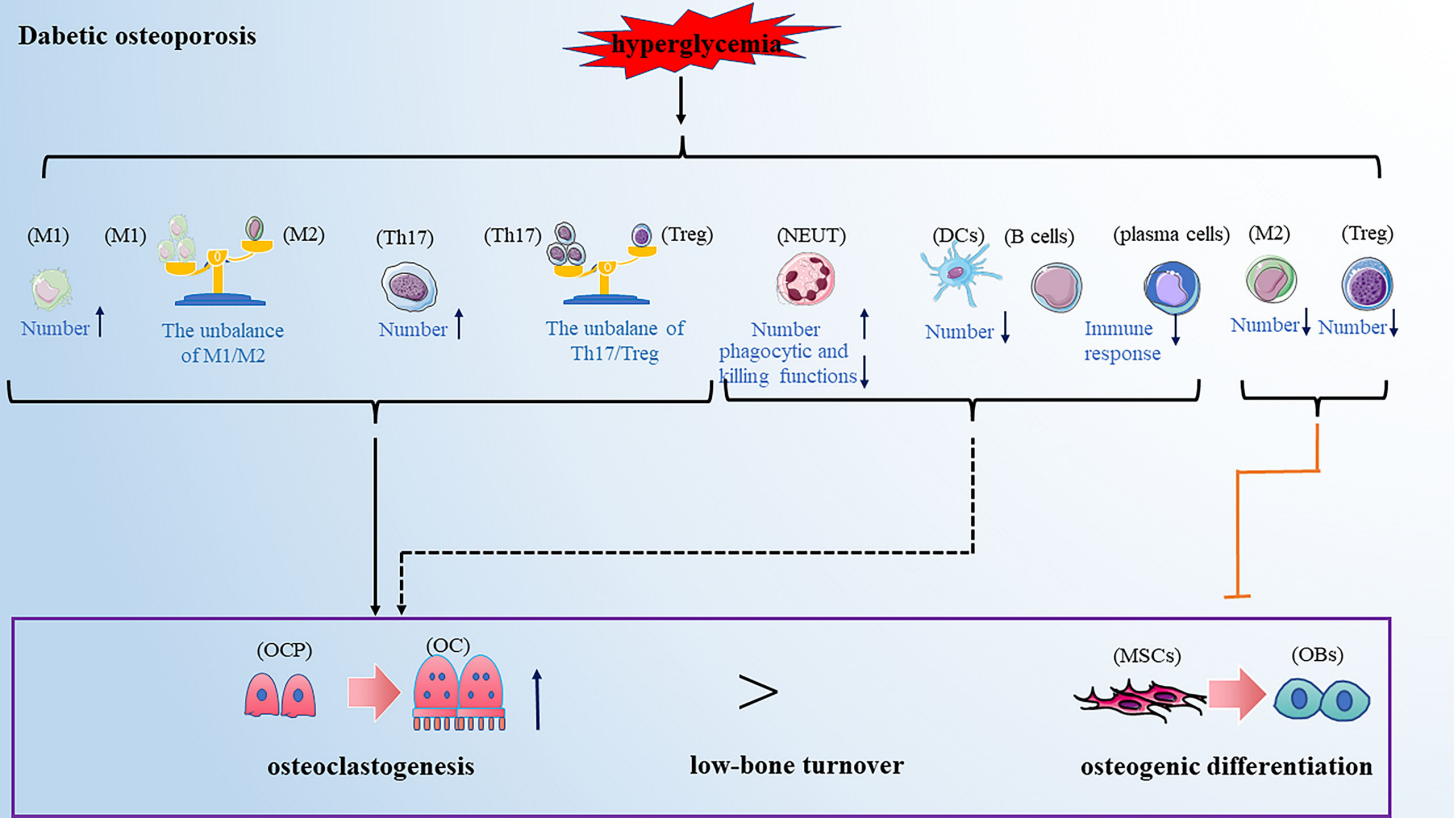
Dibetic osteoporosis is also a low-bone turnover disease. Hyperglycemia leads to the alteration of immune cells in numbers and function, promoting osteoclastogenesis and inhibiting osteogenic differentiation, partially resulting to the development of diabetic osteoporosis. Note: Various types of immune cell image material are from https://smart.servier.com.

Diabetic osteoporosis is a type of osteoporosis secondary to diabetes mellitus. Clinical data showed that the prevalence rate of osteoporosis among T2DM patients in China was 37.8%, which is 4 to 5fold that of a non-diabetic patient (97). Diabetic osteoporosis has been one of the most common complications of diabetes, seriously affecting the patient’s quality of life. Current research has pointed out that the impairments in glucose/insulin metabolism, accumulation of advanced glycation end-products (AGEs), insufficiency of the bone microvasculature and alterations in muscle endocrine function may all be involved in the development of diabetic osteoporosis (98). With the deepening of research on the immunopathological mechanism of diabetes, the alteration of immune cells has been considered an important factor in developing diabetic osteoporosis in recent years. Here we reviewed the role of various types of immune cells in developing diabetic osteoporosis (**Figure 4**).

### 5.1 Innate immune cells and diabetic osteoporosis

#### 5.1.1 Macrophages

Animal research found that hyperglycemia increased M1 macrophage polarization and osteoclast differentiation (23), and the *in vitro* experiments supported this result. A high glucose environment promotes the polarization of M1 macrophages and inhibits the polarization of M2 macrophages *in vitro* (23). Accordingly, the phenotypic switch from M2 to M1 macrophage populations increases bone resorption. Moreover, our previous results demonstrated that the enhancement of M2 macrophage polarization relieved the symptoms of osteoporosis by promoting the osteogenic difference of MSC (99). These results indirectly suggested the important role of macrophage phenotypic switch in developing diabetic osteoporosis. Through further mechanistic studies, Zhang B et al. found that overproducing reactive oxygen species (ROS) causes the polarization of macrophages toward M1 macrophages in a high glucose environment (23). ROS is an important mediator for the activating pro-inflammatory signaling pathways such as mitogen-activated protein kinases (MAPK), signal transducer and activator of transcription 1 (STAT1), signal transducer and activator of transcription 6 (STAT6) and noncanonical nuclear factor-kappaB (NF-κB) signaling which interfere with macrophage differentiation (100). Moreover, excessive glucose can alter energy metabolisms, such as increased glycolysis and mitochondrial dysfunction, producing ROS (101). Additionally, through *vivo* and *in vitro* analyses, Hu J et al. found that hyperglycemia-mediated epigenetic changes affect macrophage polarization, such as long noncoding RNA (102). Hence, those investigations will provide new targets for treating diabetic osteoporosis in an immune manner.

#### 5.1.2 Neutrophils and DCs

Previous research found that hyperglycemia increases the number of circulating neutrophils (103). However, the later studies found that the function of neutrophils is impaired in high glucose conditions, and their phagocytic and killing functions are inhibited to a certain extent (104, 105). These results reflected that hyperglycemia induces massively impaired neutrophils in peripheral blood, resulting in chronic inflammation. In addition, it has been shown that hyperglycaemia can impair the differentiation of DCs, causing a decrease in the number of DCs (106). However, there was limited evidence on the role of neutrophils and DCs in developing diabetic osteoporosis, which needs further exploration.

### 5.2 Adaptive immune cells and diabetic osteoporosis

#### 5.2.1 T cells

It has been shown that the CD4^+^ number differs in patients with diabetes combined with osteoporosis compared to patients with diabetes alone, inflecting the altered T cell subsets may be involved in developing diabetic osteoporosis. A recent review suggested that hyperglycemia induces the expansion of pro-inflammatory CD4^+^ T cells, such as Th1 and Th17 cells, and decreases the number of Treg cells (107). Accordingly, these active pro-inflammatory T cells promote bone resorption and anti-inflammatory T cells and have a bone-protective role, suggesting that hyperglycemia-mediated alteration of T cells plays a vital role in developing diabetic osteoporosis. However, the effect of high glucose on the CD8^+^ T-cells function is controversial and needs further exploration (108, 109).

#### 5.2.2 B cells

As an essential antigen-presenting cell, B cells were reported to promote inflammation in type 2 diabetes (T2DM) by regulating the T-cell function (110). Interestingly, in the analysis of cytokines from patient samples by Ip B et al., B cells supported Th17 inflammation in T2DM but not in the control group (111). These results indicated that B cells play a vital role in systemic inflammation of type 2 diabetes mellitus, providing a new insight for exploring the pathogenesis of diabetic osteoporosis. However, the previous study by Sakowicz-Burkiewicz M et al. showed that hyperglycemia could impair the function of B cells such as reducing immunoglobulin production, leading to the dysfunction of humoral immune responses (112). Unfortunately, the role of B cells in the pathogenesis of diabetic osteoporosis is relatively unclear and requires more detailed study.

## 6 Discussion

In this review, we highlighted the role of immune cells in the development of different types of osteoporosis. These results suggested that immune cells play various roles under the action of different pathogenic factors, such as estrogen deficiency, immunosenescence and diabetes. In addition, the immune cells were involved in developing drug-induced osteoporosis, such as glucocorticoid-induced osteoporosis and chemotherapy drug-induced osteoporosis. A recent study demonstrated that glucocorticoid-induced osteoporosis could not be induced in T cell-deficient mice. However, it could be re-established by transferring the splenic T cells from wide-type mice, inflecting the essential role of T cells in the development of glucocorticoid-induced osteoporosis (113). Further, some studies found that glucocorticoids promoted the accumulation of T cells in the bone marrow and these bone marrow T cells expressed high steady-state levels of RANKL, resulting osteoporosis (113). Moreover, cyclophosphamide, a chemotherapy drug, was reported to cause immunosuppression and osteoporosis. Improving the functional status of immune cells alleviates the symptoms of osteoporosis in the immunosuppressive mouse model induced by cyclophosphamide (114), suggesting the function of immune cells may be an important factor in cyclophosphamide-induced osteoporosis.

**Figure 5:**
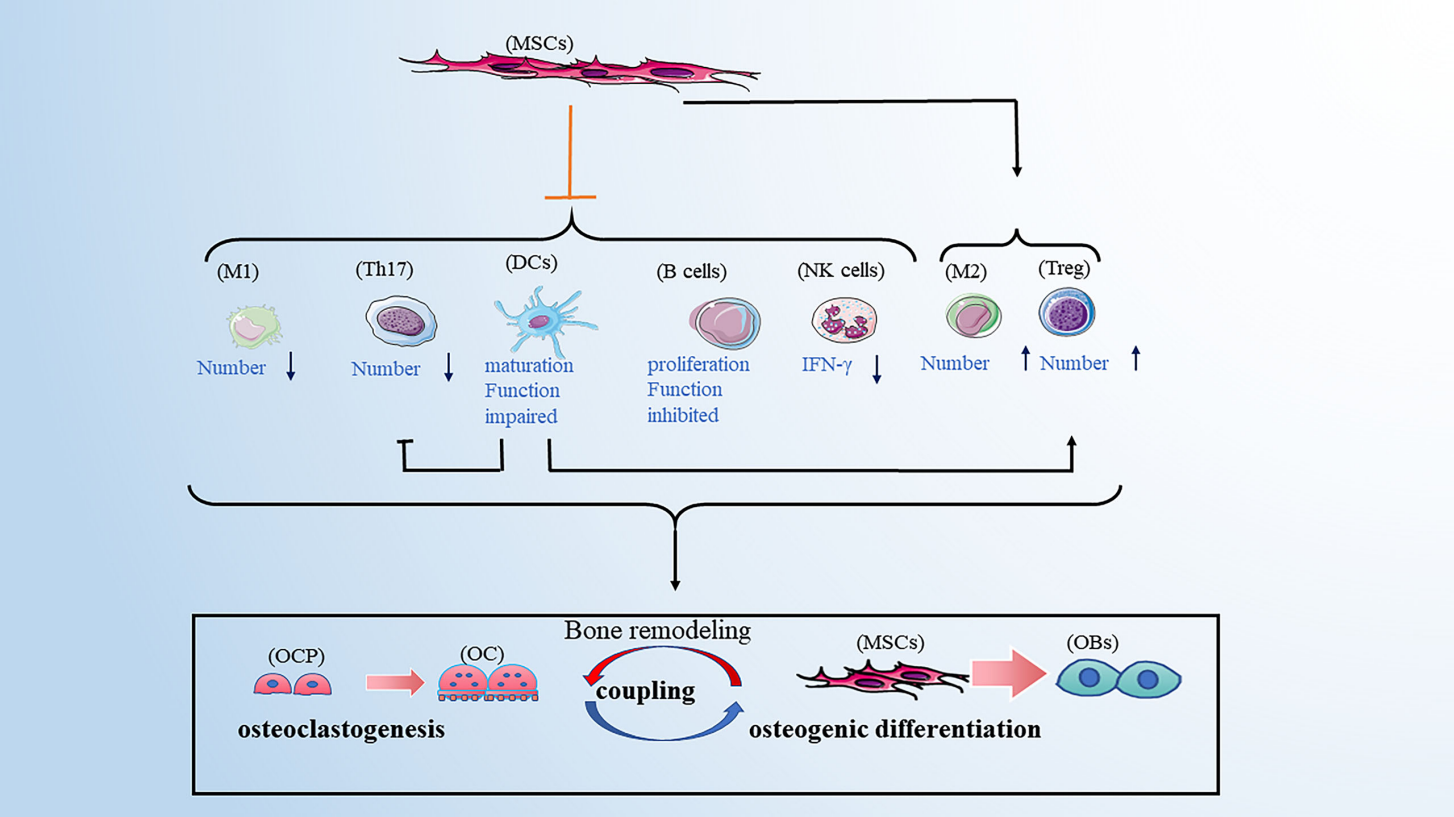
BMSCs have immunoregulatory function by affecting the immune cells. BMSCs can favor the balance of M1/M2 toward M2 macrophages, the balance of Th17/Treg toward Treg cells. BMSCs can also inhibit the maturation and function of DCs, further suppressing the activation and proliferation of T cells. BMSCs have an inhibitory role in the secretion of IFN-γ from NK cells. Moreover, BMSCs inhibit the proliferation and function of B cells. Note: Various types of immune cell image material are from https://smart.servier.com.

This review mainly discusses how immune cells affect the process of bone remodeling under different pathological conditions, which provides new insight into osteoporosis. However, in addition to differentiating into osteoblast line cells, bone marrow-derived mesenchymal stem cells (BMSCs) were shown to have an immunoregulatory function by modulating immune responses *via* cell contact-dependent or paracrine mechanisms (115). Regarding non-specific immunity, BMSCs can induce a shift in macrophages from an M1 to M2 phenotype (116), and the interaction between BMSCs and macrophages contributes to the restriction of inflammation (117). Although BMSCs can not affect the proliferation of NK cells, they reduce the IFN-γ production of NK cells (118). It was also reported that BMSCs inhibit the maturation and function of DCs, further suppressing the activation and proliferation of T cells (119). Regarding specific immunity, studies have shown that BMSCs can inhibit the proliferation of CD4^+^ and CD8^+^ T cells which mechanisms may include direct cell-cell contact, the release of soluble factors, and induction of Treg cells (120). Thus, the balance of Th1/Th2 and Th17/Treg cell phenotypes is altered. In the past, the regulatory effect of BMSCs on B cells was unclear. However, a recent study showed that BMSCs inhibit the proliferation and function of B cells (121, 122). Combined with the functional alteration of immune cells on bone remodeling described above, the immunoregulation of BMSCs is involved in the process of bone remodeling. Meanwhile, the disordered immunoregulation of BMSCs was considered to play an important role in the pathogenesis of osteoporosis (123). This provides us with a new idea for treating of osteoporosis (**Figure 5**).

To sum up, understanding the relationship between immune cells and the bone remodeling process is required to evaluate the pathological mechanism of osteoporosis. From this, identifying the immune checkpoints may provide an excellent opportunity to develop valuable immunotherapies for osteoporosis patients. However, we only focused on a few types of immune cells in this review, and more immune cells need further attention. In addition, the function of the immune system requires the participation of various cells, and different immune cells cannot function in isolation. It is hoped that future studies will pay more attention to the interaction of different types of immune cells in osteoporosis pathogenesis, which will shed much light on the role of immune cells in the development of osteoporosis.

## Author contributions

WZ, RG, XR,SZ, YC collected the data and wrote the paper, WZ and SZ drew the pictures, HL and ML designed, reviewed and edited the paper. WZ and RG contributed equally to this work and are co-first-authors. All authors contributed to the article and approved the submitted version.

## Funding

This study was partially supported by the National Natural Science Foundation of China (No. 81972072) to ML, the National Natural Science Foundation of China (No. 81800982) and the Construction Engineering Special Fund of “Taishan Young Scholars” of Shandong Province (No. tsqn202103177) to HL.

## Conflict of interest

The authors declare that the research was conducted in the absence of any commercial or financial relationships that could be construed as a potential conflict of interest.

## Publisher’s note

All claims expressed in this article are solely those of the authors and do not necessarily represent those of their affiliated organizations, or those of the publisher, the editors and the reviewers. Any product that may be evaluated in this article, or claim that may be made by its manufacturer, is not guaranteed or endorsed by the publisher.
